# Plasma sphingolipids and depressive symptoms in coronary artery disease

**DOI:** 10.1002/brb3.836

**Published:** 2017-09-26

**Authors:** Adam Dinoff, Mahwesh Saleem, Nathan Herrmann, Michelle M. Mielke, Paul I. Oh, Swarajya Lakshmi Vattem Venkata, Norman J. Haughey, Krista L. Lanctôt

**Affiliations:** ^1^ Neuropsychopharmacology Research Group Hurvitz Brain Sciences Program Sunnybrook Research Institute Toronto ON Canada; ^2^ Department of Pharmacology and Toxicology Faculty of Medicine University of Toronto Toronto ON Canada; ^3^ Department of Psychiatry Faculty of Medicine University of Toronto Toronto ON Canada; ^4^ Department of Neurology Mayo Clinic Rochester MN USA; ^5^ Department of Health Sciences Research Mayo Clinic Rochester MN USA; ^6^ Division of Clinical Pharmacology Sunnybrook Health Sciences Centre Toronto ON Canada; ^7^ Toronto Rehabilitation Institute Rumsey Centre Cardiac Rehabilitation Toronto ON Canada; ^8^ Department of Neurology Johns Hopkins University School of Medicine Baltimore MD USA; ^9^ Department of Psychiatry Johns Hopkins University School of Medicine Baltimore MD USA

**Keywords:** coronary artery disease, depressive symptoms, sphingolipid

## Abstract

**Background:**

Depression is highly prevalent in individuals with coronary artery disease (CAD) and increases the risk of future cardiac events and mortality. Sphingolipids have been implicated in the pathophysiology of both CAD and depression. This study assessed the association between plasma sphingolipid concentrations and depressive symptoms in CAD subjects.

**Methods:**

Depressive symptoms were measured using the depression subscale of the self‐reported Hospital Anxiety and Depression Scale (HADS). Sphingolipid concentrations were measured from fasting plasma samples using high‐performance liquid chromatography‐coupled electrospray ionization tandem mass spectrometry (LC/MS/MS). Linear regression models were used to assess associations between log‐transformed concentrations of plasma sphingolipids and depressive symptoms.

**Results:**

A total of 111 CAD patients (mean (*SD*) age = 63.6 ± 6.4, 84.7% male) were included. In linear regression analyses, higher plasma concentrations of ceramides C16:0 (β = 0.204, *p* = .026) and C18:0 (β = 0.209, *p* = .023) and sphingomyelin SM18:1 (β = 0.210, *p* = .024) were significantly associated with higher HADS depression subscale score after adjusting for covariates.

**Conclusion:**

Sphingolipids, in particular the ceramide species C16:0 and C18:0 and the sphingomyelin species SM18:1, may be implicated in the pathophysiology of depression in CAD. The association between plasma sphingolipid concentrations and depression should be further examined in CAD patients and in other populations.

## 1 INTRODUCTION

Coronary artery disease (CAD), defined by a narrowing of one or more coronary arteries and reduced blood flow due to build‐up of fatty plaque deposits in the arterial wall, represents a significant global health burden (Hansson, 2005; Unsar, Sut, & Durna, 2007). In addition to cardiac symptoms, it is estimated that 15%–20% of patients with CAD meet criteria for major depressive disorder (MDD) (Carney & Freedland, 2017; Hare, Toukhsati, Johansson, & Jaarsma, 2014; Rudisch & Nemeroff, 2003), a prevalence 3–4 times greater than that in the general population (Hare et al., 2014; Marcus, Taghi Yasamy, van Ommeren, Chisholm, & Saxena, 2012). Furthermore, it has been reported that an additional 30%–45% of CAD patients suffer from clinically significant depressive symptoms (Rudisch & Nemeroff, 2003). Depression is an important comorbidity of CAD because it worsens prognosis and health‐related quality of life (Bradley & Rumsfeld, 2015; Carney & Freedland, 2017; Celano & Huffman, 2011; Lichtman et al., 2014). CAD patients with depressive symptoms have higher cardiac mortality rates (Carney & Freedland, 2017; Celano & Huffman, 2011; Rudisch & Nemeroff, 2003) and a greater risk of subsequent cardiac events (van Melle et al., 2004; Whooley et al., 2008). Depression is an important comorbidity of CAD and must be addressed to reduce the major disease burden of CAD.

Current treatment options for depression in CAD include pharmacotherapy, psychotherapy, and physical activity (Lichtman et al., 2008), yet a large proportion of CAD patients do not respond to current interventions (Baumeister, Hutter, & Bengel, 2011; Taylor, 2008) even with multiple attempts (Souery, Papakostas, & Trivedi, 2006) increasing risk of future cardiac events (de Jonge et al., 2007). This is likely due to the heterogeneity in etiology and pathophysiology of depression (Belmaker & Agam, 2008; Chekroud et al., 2017; Fried & Nesse, 2015) reflected in different biological correlates such as inflammatory markers, lipid concentrations, and hypothalamic–pituitary axis abnormalities (Lamers et al., 2013). In the context of vascular diseases such as CAD, a “vascular depression hypothesis” has been proposed that postulates cerebrovascular damage may contribute to depression and depressive symptoms, especially in late life (Alexopoulos et al., 1997a). Depression with underlying vascular pathology may feature different clinical presentations than depression without underlying vascular pathology (Alexopoulos et al., 1997b; Krishnan, Hays, & Blazer, 1997). Understanding the mechanisms of depression in CAD may suggest alternative or adjunctive therapeutic strategies, potentially resulting in the creation of novel antidepressant classes.

A potential novel therapeutic target for the treatment of depression in CAD is sphingolipid metabolism (Jernigan et al., 2015; Kolesnick, 2002; Kornhuber, Muller, Becker, Reichel, & Gulbins, 2014). Sphingolipids are a diverse class of lipids containing a sphingoid base attached to an acyl group such as a fatty acid (Merrill, Sullards, Allegood, Kelly, & Wang, 2005). They are the most common form of lipid in brain cell membranes (Merrill et al., 2005; Muller et al., 2015). Lipid families in the sphingolipid class include sphingomyelins, ceramides, sphingosines, cerebrosides, and gangliosides (Merrill et al., 2005). Aberrations in sphingolipid concentrations have been reported in individuals with CAD (Huynh, 2016; Laaksonen et al., 2016; Park, Lee, Shin, & Hwang, 2015). For example, Sattler et al. found elevated plasma sphingosine‐1‐phosphate (S1P) concentrations in individuals with stable CAD after normalization to HDL‐cholesterol levels in plasma (Sattler et al., 2010). Furthermore, Deutschman et al. (2003) found an association between serum S1P concentration and severity of stenosis in CAD patients undergoing coronary angiography. Contrary to these findings, Argraves et al. (2011) observed reduced HDL content of S1P and ceramide C24:1 in individuals with coronary artery disease. Nonetheless, a difference in these sphingolipid concentrations was still found in CAD. It has been observed that human atherosclerotic lesions are enriched with sphingomyelin and ceramide species (Edsfeldt et al., 2016; Schissel et al., 1996). Sphingomyelin is transported into arterial walls by atherogenic lipoproteins and subsequently hydrolyzed into ceramides via arterial wall sphingomyelinases (SMases) (Bismuth, Lin, Yao, & Chen, 2008). Indeed, increased plasma sphingomyelin and ceramide concentrations have been shown to be independent predictors of CAD in humans (Jiang et al., 2000; Xu et al., 2016).

Altered sphingolipid concentrations have recently been reported in depression (Demirkan et al., 2013; Gracia‐Garcia et al., 2011; Liu et al., 2016). Elevations in ceramide concentrations have been reported in those with depression (Gracia‐Garcia et al., 2011), while sphingolipid concentrations have been observed to be associated with depressive symptoms in some populations (Demirkan et al., 2013; Liu et al., 2016; Xing et al., 2016). To our knowledge, no study to date has assessed plasma sphingolipids as correlates of depressive symptoms in a CAD population. In this study, we evaluate the association between various sphingolipid species, including ceramides and sphingomyelins, and depressive symptoms in a sample of CAD patients. As there is a high prevalence of depression in CAD, and altered sphingolipid metabolism has been associated with both disorders, CAD patients may represent an ideal patient population to further explore the relationship between sphingolipids and depression.

## 2 METHODS

### 2.1 Participants

This research was carried out in accordance with the latest version of the Declaration of Helsinki. All study procedures were approved by the Research Ethics Boards (REB) of both Sunnybrook Health Sciences Centre and University Health Network. REB‐approved informed consent was obtained from all participants before any assessments were performed. CAD patients were recruited from a cardiac rehabilitation program. Patients between the ages of 50 and 75 entering cardiac rehabilitation were asked to participate in this study. All study participants were required to have one or more of the following as evidence of CAD: coronary angiographic evidence of ≥50% blockage in ≥1 major coronary artery, previous hospitalization for acute MI, a prior revascularization procedure (stent or CABG), or previous diagnosis of ischemic heart disease. Only participants with stable CAD, as defined by no hospitalizations for cardiac events within the 4 weeks prior to participation in the study, were included the study. All participants were required to be taking a statin medication. This inclusion criterion was implemented in order to standardize the study population, as statins are the standard of care in CAD and may impact sphingolipid concentrations (Ng et al., 2014; Wei et al., 2013) as well as mood and cognition (Kim et al., 2015; Power, Weuve, Sharrett, Blacker, & Gottesman, 2015).

Similarly, use of antidepressants within 3 months of study participation constituted an exclusion criterion due to the impact of some antidepressants on ceramide and other sphingolipid concentrations (Gulbins et al., 2013, 2015; Kornhuber et al., 2014). Some antidepressants, including many SSRIs and tricyclic antidepressants (TCAs), have been shown to inhibit acidic sphingomyelinase (aSMase), an enzyme responsible for catalyzing the breakdown of sphingomyelin to ceramide and phosphorylcholine, resulting in decreased ceramide concentrations (Kornhuber et al., 2010). Use of antipsychotics was also an exclusion criterion due to their effects on mood and cognitive outcomes, as well as their potential effects on sphingolipid concentrations (Alexander, Gallagher, Mascola, Moloney, & Stafford, 2011; Hori et al., 2015; Kornhuber, Tripal, Gulbins, & Muehlbacher, 2013; Kornhuber et al., 2008). Patients diagnosed with any dementia, Parkinson's disease, Huntington's chorea, an autoimmune disease (e.g., multiple sclerosis, rheumatoid arthritis, Crohn's disease), active cancer, impaired liver or kidney function, or history of cerebrovascular accident (CVA), transient ischemic attack (TIA), brain tumor, subdural hematoma, epilepsy, or any other conditions likely to significantly impact mood or cognition were excluded from this study. Furthermore, patients with significant cognitive impairment (as assessed by a standardized mini mental state examination [sMMSE] of ≤24), bipolar disorder, or schizophrenia were also excluded.

### 2.2 Assessments

Presence and severity of depressive symptoms were measured using the depression subscale of the Hospital Anxiety and Depression Scale (HADS‐D). The Hospital Anxiety and Depression Scale is a 14‐item self‐reported scale with integrated depression and anxiety subscales (Zigmond & Snaith, 1983). It was created for use in a medical outpatient population (such as the present study population) and has been validated for use in CAD populations (Martin, Lewin, & Thompson, 2003; Zigmond & Snaith, 1983). Presence of MDD or minor depression was assessed using a structured clinical interview for the diagnosis of *DSM–IV* criteria depression (SCID; First, Spitzer, Gibbon, & Williams, 2002; Lobbestael, Leurgans, & Arntz, 2011). Participants were classified as depressed if either minor or major depression was present. Cardiac medical history and other relevant medical and surgical history were obtained from study participants and via electronic and physical medical records. Cardiopulmonary and metabolic health indices (VO_2_ peak, resting HR, max HR, BP, height, weight, BMI, and body fat percentage) were also collected from electronic medical records. CAD severity was indicated by the total number of stenosed major coronary arteries as well as the sum of the percent blockages of all major coronary arteries (cumulative stenosis). Demographic information such as age, gender, marital status, employment status, and socioeconomic status as well as smoking history were obtained verbally from study participants.

### 2.3 Plasma sphingolipid measurements

A 12‐hr fasting blood sample was collected from each participant. The majority of participants were scheduled to come for assessments and blood collection at 09:00 ± 1 hr. This standardized time and fasting blood allowed for relative control of diurnal and dietary influences on ceramide and other lipid concentrations. Blood was drawn into EDTA containing vacutainer tubes in order to collect plasma samples. Blood was then centrifuged at 1,000 *g* for 15 min, and plasma was immediately isolated, aliquoted, and stored at −80°C until analyzed.

Forty‐five different sphingolipid species (for full list of species, see Appendix A) were measured including nine ceramide species, ten sphingomyelin species, sphingosine, sphingosine‐1‐phosphate (S1P), and a number of monohexyl and lactosyl ceramides. Sphingolipid species were quantified using high‐performance liquid chromatography‐coupled electrospray ionization tandem mass spectrometry (HPLC ESI‐MS/MS). All measurements were performed blind to clinical characteristics of the participants. Plasma samples were injected into a PerkinElmer HPLC (Waltham, MA, USA) equipped with a phenomenex, luna 100 × 2 mm, 5 μm, C18 column coupled with guard column containing identical packaging material (Phenomenex, Torrance, CA, USA) using a PAL autosampler. A mobile phase consisting of 85% methanol, 15% H_2_O, and 5 mmol/L ammonium formate was first injected for 0.5 min into the LC column for pre‐equilibration. The column was then eluted with a second mobile phase consisting of 99% methanol, 1% formic acid, and 5 mmol/L ammonium formate at a flow rate of 100.0 μl/min. The eluted sample was then injected into the ion source for detection and quantification of sphingolipid species (*m/z* 264.4, 266.4 for ceramides and 184.4 for sphingomyelins, respectively). Data were collected and processed by Analyst 1.4.2 software package. Sphingolipid concentrations were initially presented in counts per second (cps), but were converted to ng/ml by applying a standard curve. All sphingolipid measurements were done in a single laboratory blind to clinical information.

### 2.4 Statistical analyses

Plasma sphingolipid concentrations were log‐transformed to adjust for non‐normal positively skewed distributions as done previously (Gracia‐Garcia et al., 2011; Mielke et al., 2010, 2014; Saleem et al., 2013). HADS‐D scores were natural log‐transformed, after adding 1 to each score to account for the inability to transform scores of 0, to adjust for a non‐normal positively skewed distribution as has been previously done with depression rating scales (Andersen, Goyal, Westbrook, Bishop, & Carson, 2016; Kiecolt‐Glaser, Belury, Andridge, Malarkey, & Glaser, 2011; Mamalakis et al., 2006; Shah, Zonderman, & Waldstein, 2013). Associations between participant sociodemographic and clinical characteristics with HADS‐D score were assessed using Pearson's correlations and analysis of variance (ANOVA). ANOVAs were used to assess group differences in mean plasma sphingolipid concentration between participants with a documented history of depression and those without a documented history of depression. As a variable reduction strategy, bivariate analyses using Pearson's correlations were used to identify plasma sphingolipids significantly correlated with HADS‐D score. After identifying those associations significant at the *p* < .05 level, multiple linear regression models were used to assess whether higher plasma concentrations of significant species were associated with greater depressive symptoms, after adjusting for significant covariates including peak oxygen uptake (VO_2 Peak_) and number of pack‐years smoked. For subgroup analysis, participants were broken up into quintiles based on HADS‐D scores, and mean plasma concentrations of sphingolipid species were compared between the highest and lowest quintiles using analysis of covariance (ANCOVA) adjusting for VO_2 Peak_ and pack‐years smoked. Groups were divided based on quintiles as this division allowed for a cleaner division of participants in each subgroup, so that all participants in the lowest quintile had a HADS‐D score of 0. Bivariate analyses were also used to assess associations between plasma sphingolipid concentrations and measures of CAD severity. No correction for multiple comparisons was performed as these analyses were considered exploratory. All analyses were performed using IBM SPSS Version 20 (IBM Corp, Armonk, NY, USA). Data are presented as mean ± standard deviation unless otherwise stated.

## 3 RESULTS

One hundred and eleven CAD patients completed this study. Sociodemographic characteristics, cardiac risk factors, and concomitant medications are presented in Table 1. Sixteen participants had a documented history of depression. There were no significant differences in mean plasma concentration of any of the sphingolipids measured in the study between participants with a history of depression and those without a documented history of depression. Fifteen participants had a history of antidepressant use. Of these fifteen participants, seven had not taken antidepressants in the previous five years leading up to the study. Seven participants were exposed to antidepressants for less than three months total, while three participants were on the medication for longer than a year. Nineteen participants were diabetic. Ninety‐six percent of participants were taking a platelet inhibitor at the time of the study and seventy‐eight percent of participants were taking a beta‐blocker. Use of calcium channel blockers and diuretics were infrequent.

**Table 1:** Participant sociodemographic and clinical characteristics (*n* = 111)

Characteristic	Mean ± *SD* or *n* (% of study population)	Association with HADS‐D score	
		*F* or *r*	*p* value
Age	63.6 ± 6.4	0.024	.800
Gender (% male)	94 (84.7)	0.174	.677
Ethnicity (% Caucasian)	93 (83.8)	0.539	.464
Years of education	16 ± 3	−0.130	.174
Cardiovascular risk factors			
BMI	29.2 ± 5.0	0.077	.425
Pack‐years smoked	14.7 ± 23.6	0.225	.017*
Resting systolic BP	125.6 ± 17.6	−0.018	.851
Resting diastolic BP	77.2 ± 9.6	−0.022	.821
Total cholesterol	3.5 ± 0.9	−0.024	.799
Diabetes	19 (17.1)	0.022	.881
Hypertension	68 (61.3)	0.001	.977
Cardiac history			
MI	53 (47.7)	0.017	.896
CABG	38 (34.2)	0.003	.956
PTCA	73 (65.8)	0.021	.886
CAD severity			
Number of stenosed major coronary arteries^a^	2.0 ± 0.8	0.084	.407
Cumulative stenosis (sum of % blockages)^b^	151.4 ± 69.1	−0.028	.796
Cardiorespiratory fitness			
VO_2 Peak_	20.9 ± 5.6	−0.281	.003*
Max HR	122.4 ± 20.4	−0.167	.079
Medication use			
Beta‐blocker	87 (78.4)	0.473	.493
Calcium channel blocker	16 (14.4)	0.322	.572
Diuretic	19 (17.1)	0.036	.851
Antihypertensive	80 (72.1)	0.983	.324
Platelet inhibitor	107 (96.4)	0.082	.775
Statin	111 (100)	NA	NA
Antidepressant	0 (0)	NA	NA
Depressive symptoms			
HADS‐D score	2.6 ± 2.5	NA	NA

BMI, body mass index; BP, blood pressure; MI, myocardial infarction; CABG, coronary artery bypass graft; PTCA, percutaneous transluminal coronary angioplasty; VO_2 Peak_, maximal oxygen consumption; HR, heart rate; CESD, Center for Epidemiological Studies Depression Scale; NA, not applicable; *SD*, standard deviation.

a Eleven participants were missing data on the number of stenosed major coronary arteries.

b Twenty participants were missing data on cumulative stenosis.

*p < .05.

Of the demographic characteristics, VO_2 Peak_ and number of pack‐years smoked were significantly associated with total HADS‐D score (Table 1). Of the forty‐five different sphingolipid species measured in this study, three ceramide species, C16:0, C18:0, and C22:1, and two sphingomyelin species, SM18:0 and SM18:1, were significantly associated with total HADS‐D score in bivariate analysis (Table 2). There were no differences in mean plasma concentrations of these species between participants with and without a documented history of depression (C16:0, *F*_1,109_ = 1.30, *p* = .26; C18:0, *F*_1,109_ = 0.01, *p* = .92; C22:1, *F*_1,109_ = 0.46, *p* = .50; SM18:0, *F*_1,109_ = 0.14, *p* = .71; SM18:1, *F*_1,109_ = 0.03, *p* = .86).

**Table 2:** Bivariate associations between significant sphingolipid species and natural log‐transformed HADS‐D score

Sphingolipid	*r*	*p* value
C16:0	.246	.009*
C18:0	.258	.006*
C22:1	.191	.044*
SM18:0	.204	.032*
SM18:1	.243	.010*

*p < .05.

In linear regression analysis, adjusting for VO_2 Peak_ and pack‐years smoked, C16:0 concentration (β = 0.204, *p* = .026), C18:0 concentration (β = 0.209, *p* = .023), and SM18:1 concentration (β = 0.210, *p* = .024) remained significantly associated with HADS‐D score (Tables 3 and 4). C22:1 and SM18:0 concentrations were not significant predictors of HADS‐D score after adjusting for VO_2 Peak_ and pack‐years smoked. Neither measure of CAD severity (cumulative stenosis and number of stenosed major coronary arteries) was associated with any of the sphingolipid species measured in this study (data not shown).

**Table 3:** Overall model statistics of linear regression analyses

	Adjusted *R* ^2^	*F*	*df*	*p* value
Model with C16:0	0.121	6.06	110	.001*
Model with C18:0	0.123	6.12	110	.001*
Model with SM18:1	0.122	6.11	110	.001*

*p < .05.

**Table 4:** Model parameters of linear regression analyses

Model	Predictor	β	*p* value
C16:0	C16:0	0.204	.026*
	VO_2 Peak_	−0.218	.021*
	Pack‐years smoked	0.150	.109
	Constant	—	.727
C18:0	C18:0	0.209	.023*
	VO_2 Peak_	−0.215	.023*
	Pack‐years smoked	0.144	.123
	Constant	—	.003*
SM18:1	SM18:1	0.210	.024*
	VO_2 Peak_	−0.192	.045*
	Pack‐years smoked	0.183	.051
	Constant	—	.091

*p < .05.

In subgroup analysis, participants in the highest quintile of HADS‐D score (mean score ± *SD* = 6.83 ± 1.59, range: 5–11) had significantly greater mean plasma concentrations of C16:0 (*F*_1,42_ = 11.31, *p* = .002), C18:0 (*F*_1,42_ = 8.97, *p* = .005), and monohexylceramide C18:0 (*F*_1,42_ = 6.23, *p* = .017) after adjusting for VO_2 Peak_ and pack‐years smoked compared to those in the lowest quintile (HADS‐D score = 0). Mean plasma concentrations of the remaining sphingolipid species measured in this study were not significantly different between these two quintiles.

## 4 DISCUSSION

We analyzed the relationships between numerous sphingomyelins, ceramides, glucosylceramides, and sphingosine and depressive symptoms in a CAD population. Results from this study suggest that certain sphingolipid species, namely the ceramides C16:0 and C18:0 and the sphingomyelin SM18:1, may be associated with depressive symptoms in CAD patients. These findings suggest a possible role of these species in the pathophysiology of depression in CAD. Given the high incidence of CAD combined with the high prevalence of comorbid depressive symptoms, and the impact of treatment‐resistant depressive symptoms on prognosis, this is an important finding.

Our findings are consistent with previous reports in other populations. Both ceramide species C16:0 and C18:0 have been implicated in depression in previous studies (Demirkan et al., 2013; Gracia‐Garcia et al., 2011). Gracia‐Garcia et al. observed elevated plasma concentrations of these ceramide species in depressed individuals with and without Alzheimer's disease (Gracia‐Garcia et al., 2011). In a Dutch family‐based lipidomics study, Demirkan et al. found an association between ceramide C18:0 and depressive symptom severity in their study population (Demirkan et al., 2013). Importantly, both species have been implicated in apoptosis (Babenko, Hassouneh, Budvytiene, Liesiene, & Geilen, 2010; Cremesti et al., 2001; Grassme et al., 2001; Senkal et al., 2007; Thomas, Matsko, Lotze, & Amoscato, 1999). For ceramide C16:0, concentrations were found to be elevated in apoptotic cells within 2 hr of being exposed to ionizing radiation (Thomas et al., 1999). Moreover, C16:0 has been shown to be required for the initiation of Fas‐induced apoptosis and may act as a second messenger after Fas ligation by inducing Ras activation and subsequent apoptosis (Cremesti et al., 2001; Grassme et al., 2001; Gulbins et al., 1995). C18:0 has been observed to induce apoptosis of keratinocytes in a concentration‐dependent manner (Babenko et al., 2010). In addition, concentrations of C18:0 were found to be elevated in chemotherapy‐induced apoptosis (Senkal et al., 2007). As both C16:0 and C18:0 may be proapoptotic, it is feasible that elevated concentrations of these species may contribute to neurodegeneration in depression. In addition, increased ceramide concentrations may contribute to pathophysiological mechanisms of depression by inducing intracellular production of reactive oxygen species, thus enhancing oxidative stress (Andrieu‐Abadie, Gouaze, Salvayre, & Levade, 2001; Phillips, Allen, & Griffiths, 2002). Indeed, increased oxidative stress has been reported in patients with MDD (Sarandol et al., 2007; Stefanescu & Ciobica, 2012). Furthermore, ceramides may alter the reuptake of monoamine neurotransmitters, resulting in reduced serotonergic transmission, a pathophysiological hallmark of depression (Ressler & Nemeroff, 2000; Riddle, Rau, Topham, Hanson, & Fleckenstein, 2003).

With regard to SM18:1, there is little existing research on its potential contribution to depression or other psychiatric disorders. SM18:1 makes up a small component of brain cell membranes, accounting for approximately 2%–5% of total sphingomyelin content (O'Brien & Rouser, 1964; O'Brien & Sampson, 1965). Interestingly, SM18:1 content appears to increase with age (O'Brien & Sampson, 1965), suggesting it may be involved in processes associated with brain aging. It is a ceramide precursor and as such, it is well positioned to influence C16:0 and C18:0. Of note, none of the significant sphingolipid species identified in this study were associated with measures of CAD severity in this population. CAD severity was not significantly associated with HADS‐D score either.

If in fact certain ceramide species and other sphingolipids do contribute to depression pathophysiology, novel pharmacotherapies aimed at reducing ceramide concentrations may be an effective strategy in treating depression and/or depressive symptoms in those with or without CAD. Already, candidate drugs that reduce ceramide concentrations exist (Cinar et al., 2014; Kornhuber et al., 2010; Miyake, Kozutsumi, Nakamura, Fujita, & Kawasaki, 1995). A term for functional inhibitors of acidic sphingomyelinases, FIASMAs, created by Kornhuber et al., reflects drugs that reduce ceramide concentrations by inhibiting aSMase (Kornhuber et al., 2010). Many FIASMAs have other primary pharmacological targets aside from aSMase and are approved for use in humans to treat various diseases (Kornhuber et al., 2010).

The findings from this study must be considered preliminary as we did not correct for multiple comparisons. Multiple comparisons were not corrected for as these analyses were considered exploratory and hypothesis generating. Forty‐five different sphingolipid species were explored as correlates of depressive symptoms in CAD patients and only three species were found to be significantly associated with depressive symptom severity after adjusting for significant covariates. However, two of those species have been identified as associated with depression previously.

A further limitation of the present study is that although antidepressant use within the three months prior to study participation constituted an exclusion criterion, it is possible that antidepressant use prior to this time period may have affected sphingolipid concentrations in study participants. As mentioned, fifteen study participants had previous exposure to an antidepressant medication; however, only one of these participants had a documented use of antidepressants within one year prior to study participation. Moreover, two of these participants were only exposed to very low doses of antidepressant medication prescribed as a sleeping aid. It should also be noted that most patients in the present study did not meet criteria for MDD. Several participants had a documented history of depression, but at the time of the study most participants were not in the midst of a depressive episode. Nonetheless, the prevalence of depressive symptoms in this study population was representative of the general CAD population (Lichtman et al., 2008; Swardfager et al., 2011). While this makes the study highly relevant to the CAD population, where subsyndromal symptoms predominate, these findings may not apply to those with MDD. Depressive symptoms and subthreshold depression in CAD have been shown to be clinically important, as they predict mortality (Barefoot et al., 2000) and adherence to treatment (Gehi, Haas, Pipkin, & Whooley, 2005; Rieckmann et al., 2006; Ziegelstein et al., 2000). As such, identifying novel treatment targets is of particular importance as milder forms of depression impact prognosis in those with CAD (Barefoot et al., 2000) and are often undertreated (Ellis, Eagle, Kline‐Rogers, & Erickson, 2005). As a next step, longitudinal studies are warranted to determine if changes in ceramide concentrations reflect changes in depressive symptoms, and if an increase in ceramide concentrations is associated with an increased incidence of depression and/or depressive symptoms.

This study assessed associations between plasma sphingolipids and depressive symptoms in a sample of CAD patients. If ceramides and other sphingolipids play a role in depression, they likely exert their depressogenic effects in the brain rather than in the periphery (Kornhuber et al., 2014; Muller et al., 2015). The relationship between plasma and brain concentrations of sphingolipids has not been fully elucidated. One study in rats observed increases in both plasma and brain cortex concentrations of ceramides after injection with lipopolysaccharide (LPS), and showed that the ceramide species C6 was able to cross the rat blood–brain barrier (BBB) (Zimmermann et al., 2001). However, the rat and human BBB are structurally and functionally different and thus we cannot infer from those studies that ceramides can cross the human BBB. The current research adds to the growing body of clinical evidence that plasma sphingolipids may reflect central processes, either as by‐products of disease states in the central nervous system or as contributors to these disease states, or both (Assies et al., 2010; Demirkan et al., 2013; Hammad et al., 2012; Han et al., 2011; Mielke et al., 2011). Numerous studies have observed associations between peripheral sphingolipid concentrations and psychiatric symptoms (Demirkan et al., 2013; Mielke et al., 2013; Xing et al., 2016) as well as elevated concentrations of sphingolipids in the periphery of individuals with psychiatric disorders such as depression and posttraumatic stress disorder (Gracia‐Garcia et al., 2011; Hammad et al., 2012). Currently, little is known about the relationship between peripheral and central biomolecules such as sphingolipids. Future studies assessing relationships between peripheral and central sphingolipid concentrations are warranted to understand possible contributions of peripheral sphingolipids to central disease processes.

## 5 CONCLUSION

Findings from this study suggest that further investigation of the relationship between ceramides, in particular C16:0 and C18:0, and other sphingolipids and depressive symptoms in CAD patients is warranted. We cannot conclude from this study whether ceramides and/or other sphingolipids contribute to depression pathophysiology in those with or without CAD. If ceramides are found to be implicated in depression, novel pharmacotherapies aimed at reducing ceramide concentrations may be an effective strategy in treating depression and depressive symptoms. Novel pharmacotherapies for the treatment of depression and depressive symptoms may result in improved patient outcomes and improved health‐related quality of life for those who suffer from these illnesses. Furthermore, ceramide concentrations may one day be useful biomarkers for depression, guiding more successful diagnoses and therapies for depressed patients.

## CONFLICT OF INTEREST

None declared.

## APPENDIX1 1

### List of sphingolipid species measured in plasma

#### 1

**Table:**

Sphingolipid	Abbreviation
Ceramide 16:0	C16:0
Ceramide 18:0	C18:0
Ceramide 20:0	C20:0
Ceramide 22:0	C22:0
Ceramide 24:0	C24:0
Ceramide 26:0	C26:0
Ceramide 16:1	C16:1
Ceramide 22:1	C22:1
Ceramide 24:1	C24:1
Sphingomyelin 16:0	SM16:0
Sphingomyelin 18:0	SM18:0
Sphingomyelin 20:0	SM20:0
Sphingomyelin 22:0	SM22:0
Sphingomyelin 24:0	SM24:0
Sphingomyelin 16:1	SM16:1
Sphingomyelin 18:1	SM18:1
Sphingomyelin 20:1	SM20:1
Sphingomyelin 22:1	SM22:1
Sphingomyelin 24:1	SM24:1
Dihydrosphingomyelin 16:0	DHSM16:0
Dihydrosphingomyelin 18:0	DHSM18:0
Dihydrosphingomyelin 20:0	DHSM20:0
Dihydrosphingomyelin 22:0	DHSM22:0
Monohexylceramide 16:0	MHxC16:0
Monohexylceramide 18:0	MHxC18:0
Monohexylceramide 20:0	MHxC20:0
Monohexylceramide 22:0	MHxC22:0
Monohexylceramide 24:0	MHxC24:0
Monohexylceramide 26:0	MHxC26:0
Monohexylceramide 16:1	MHxC16:1
Monohexylceramide 22:1	MHxC22:1
Monohexylceramide 24:1	MHxC24:1
Monohexylceramide 26:1	MHxC26:1
Dihydromonohexylceramide 16:0	DHMHxC16:0
Dihydromonohexylceramide 22:0	DHMHxC22:0
Dihydromonohexylceramide 24:0	DHMHxC24:0
Lactosylceramide 16:0	LacCer16:0
Lactosylceramide 22:0	LacCer22:0
Lactosylceramide 24:0	LacCer24:0
Lactosylceramide 18:1	LacCer18:1
Lactosylceramide 24:1	LacCer24:1
Lactosylceramide 26:1	LacCer26:1
Dihydrolactosylceramide 16:0	DHLacCer16:0
Sphingosine	Sphingosine
Sphingosine‐1‐phosphate	S1P
